# A vestige of a prebiotic bonding machine is functioning within the contemporary ribosome

**DOI:** 10.1098/rstb.2011.0146

**Published:** 2011-10-27

**Authors:** Miri Krupkin, Donna Matzov, Hua Tang, Markus Metz, Rinat Kalaora, Matthew J. Belousoff, Ella Zimmerman, Anat Bashan, Ada Yonath

**Affiliations:** Weizmann Institute of Science, Rehovot 76100, Israel

**Keywords:** proto-ribosome, ribosomal symmetrical region, peptide bond formation, RNA world

## Abstract

Based on the presumed capability of a prebiotic pocket-like entity to accommodate substrates whose stereochemistry enables the creation of chemical bonds, it is suggested that a universal symmetrical region identified within all contemporary ribosomes originated from an entity that we term the ‘proto-ribosome’. This ‘proto-ribosome’ could have evolved from an earlier machine that was capable of performing essential tasks in the RNA world, called here the ‘pre-proto-ribosome’, which was adapted for producing proteins.

## 1. Introduction

Ribosomes are the universal cellular molecular machines that play the main role in the translation of the genetic code into proteins. In this process, messenger RNA (mRNA) carries the genetic information, and tRNA molecules carry the amino acids. The ribosome possesses a channel along which the mRNA chain progresses as well as three tRNA binding sites, designated as A (aminoacyl), P (peptidyl) and E (exit). Among those, decoding as well as peptide bond formation are performed by the A- and P-tRNAs.

In all organisms, ribosomes consist of two subunits of unequal size, each having defined tasks. The small ribosomal subunit is involved in the initiation of the translation process, in selecting the translated frame, in decoding the genetic message, and in controlling the fidelity of codon–anticodon interactions. The large subunit catalyses the formation of the peptide bond, elongates the newly synthesized proteins and gates the nascent chains by channelling them through their dynamic exit tunnel. The ribosomes are multi-component riboprotein assemblies of molecular weights that span between 2.5 MDa (in prokaryotes) and 4 MDa (in eukaryotes). Their main catalytic task is peptide bond formation. The fact that peptide bonds can be made spontaneously from activated amino acids, should not be overlooked. The ribosome increases the pace of this reaction, positions the substrates in the right stereochemistry for peptide bond formation and substrate-mediated catalysis, and ensures processivity.

**Figure 1.**
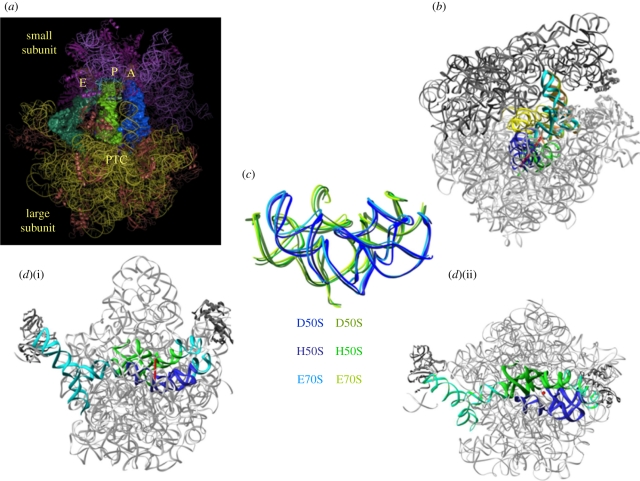
The ribosomal symmetrical region. In all panels, the ribosomal RNA is shown in grey. The blue and green ribbons indicate the ribo-phosphate ester backbones of the rRNA of the A- and the P-regions of the symmetrical region. The red rod indicates the position of the imaginary axis and the red dot indicates a section cut parallel to the direction of the symmetry axis. (*a*) An overall representation of the ribosome with its three tRNA substrates and the location of the peptidyl transferase centre (PTC). (*b*) The position of the symmetrical region within the entire ribosome, with A- and P-site tRNAs (cyan and brown, respectively), and the intersubunit bridge that connects between the rims of the cavity leading to the PTC and the decoding centre. (*c*) The universality of the symmetrical region is indicated by the superposition of the pocket suggested to represent the remnant of the proto-ribosome as in the large subunits from the eubacteria *Deinococcus radiodurans*, D50S (PDB accession code 1NJP), the archaeon *Haloarcula marismortui*, H50S (PDB accession code 1VQN) and the entire ribosome from *Escherichia coli* E70S (PDB accession code 2AVY). (*d*) Two views (i) and (ii) of the symmetrical region and its non-symmetrical extensions (in cyan) within the contemporary ribosome, which connect all ribosomal functional regions.

The L-shaped tRNA molecules are composed primarily of double helices, with an anticodon stem loop at one edge and a single-stranded universal moiety composed of the three nucleotides cytosine, cytosine, adenine (CCA) on their other edge (their 3′ end). The CCA end of the A-site tRNA is aminoacylated with the amino acid to be incorporated into the nascent protein. Similarly, the newly formed polypeptide chains are bound chemically to the CCA end of the P-site tRNA. The mRNA and the anticodon loop of the tRNA molecules interact with the small subunit, whereas the acceptor stem and the 3′ end of the tRNA molecules bind to the large subunit. Hence, all three tRNA sites span the two subunits, and each of the three tRNA molecules is located on both subunits (figure 1*a*). During the elongation cycle, the three tRNA molecules act in a concerted manner and translocate from the A- to the P-site and from there to the E-site. In conjunction with the tRNA translocation, the mRNA progresses by precisely one codon.

Analysis of the various high-resolution crystal structures of bacterial ribosomal particles at various functional states, as well as the slightly lower resolution structure of the eukaryotic ribosome, which have become available in the last decade [1–8], indicate that despite the size difference the ribosomes' functional regions, namely the decoding centre and the site where the peptide bonds are formed (called peptidyl transferase centre, PTC), are composed solely of ribosomal RNA (rRNA) and devoid of proteins. This is in accord with the ribosomes' composition, as they contain long RNA chains alongside many different proteins with a ratio of RNA to proteins being approximately 2 : 1, except for in the mitochondria, where RNA : proteins is 1 : 1. Sequence analysis has clearly shown that these sites are highly conserved across all kingdoms of life, including mitochondria. Hence, all ribosomes are RNA enzymes, namely ribozymes. Importantly, compared with all known ribozymes, which are rather inefficient catalysts, the ribosomes perform remarkably well. Thus, a typical prokaryotic ribosome makes 15–20 peptide bonds per second with a fidelity rate of over 1 : 10 000. The efficient operation of the ribosomes seems to be achieved by the incorporation of the uniquely shaped ribosomal proteins, which contain long C- and N-terminal tails and/or elongated internal loops, into the rRNA intricate structure, thus maintaining its accurate shape and its controlled dynamics. This ingenious design turned the typical slow RNA ribozyme to an efficient polymerase [9].

## 2. The ribosomal symmetrical region

The high-resolution structures of the bacterial ribosomes from *Deinococcus radiodurans, Haloarcula marismortui* and *Escherichia coli* [1–7] indicated that in the contemporary ribosome the PTC is situated in the centre of a universal conserved structural element. This region is arranged in a semi-symmetrical manner [10,11], an extremely unusual feature within the otherwise asymmetric ribosome. An almost identical symmetrical region exists in all known ribosome structures (figure 1) from prokaryotes [1–7] and eukaryotes [8]. Even the lower resolution structure of mitochondrial ribosomes [12] contains such a region. The structure preservation and the exceptionally high sequence conservation (over 95%) indicate that the existence of this region is beyond evolutionary stresses, and thus point to its ancient origin [13–17]. This symmetry related region is composed of 180 nucleotides, with an inner core of 120 nucleotides. Its outer 60-nucleotide shell contains the A- and P-loops, which is where the ribosome accommodates the tRNA 3′ ends. This symmetrical region connects all ribosomal functional regions (figure 1*d*), hence can provide the machinery for signal transmission between them.

Careful structural analysis of this pseudo-symmetrical region revealed that it might have been a remnant of a prebiotic machine for chemical bonding, which is still functioning within all of the contemporary ribosomes. Importantly, this twofold rotational symmetry operation relates the backbone and the nucleotide orientations of its two sub-regions, but not the RNA sequences. The existence of the symmetrical fold, regardless of the non-symmetrical sequence, indicates the superiority of function over sequence preservation.

**Figure 2.**
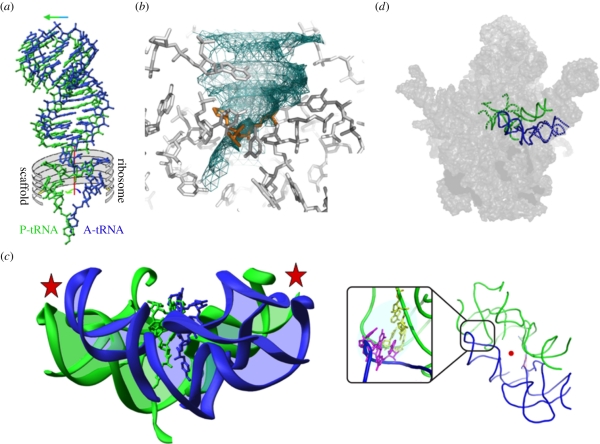
Substrate location in the pocket suggested to represent the remnant of the proto-ribosome and the proposed reaction transition state. In all panels, the A- and P-sub-regions of the symmetrical region as well as the A-site and P-site tRNAs are shown in blue and green, respectively, and the ribosome components are shown in grey. The red rod indicates the position of the imaginary symmetry axis and the red dot indicates a section cut parallel to its direction. (*a*) A cartoon representing the translocation of the A-site tRNA into the P-site. The main motion of the tRNA helical region is represented by the horizontal arrow and rotatory motion of the A-site tRNA 3′ end is represented by the curved arrow. The so-derived position of the 3′ end of the P-site tRNA is also shown (in green). The ribosome scaffold that guides the motion is represented by the grey ‘ribs’. (*b*) The volume consumed by the motion is shown as a net, obtained by snapshots of the rotatory motion of the A-site 3′ end (every 15° around the imaginary rotation axis (not shown here)). The transition state analogue that is formed during the rotary motion, just before reaching the P-site [18] is shown in orange. (*c*) The RNA backbone of the pocket suggested to represent the remnant of the proto-ribosome with the tRNAs 3′ ends, highlighting the connections between the two halves (by the red stars). The A-site substrate (blue) was obtained by cutting out (computationally) the acceptor stem from the crystal structure of complexes of the tRNA acceptor stem and the 3′ end mimic with D50S [10]. The P-site substrate was derived from the A-site amino acid by applying the rotatory motion. The box shows a GNRA tetra loop that contains the A-minor motif (the bases involved are shown in magenta and yellow). (*d*) The symmetrical region superposed on the interface surface of the large ribosomal subunits, with its inner core of 120 nucleotides shown in full line, and the shell containing the A- and P-loops shown in broken lines.

The striking architecture of the symmetrical region positions the ribosome's tRNA substrates in a favourable stereochemistry for peptide bond formation, for nascent protein elongation, for substrate-mediated catalysis and for directing the newly formed protein into its exit tunnel. It also confines the void required for the motions involved in substrate translocation within the PTC, a key component of nascent protein elongation, namely the ribosome's polymerase activity. In particular, examination of the mode of binding of a tRNA mimic showed that the bond connecting its 3′ end with the tRNA acceptor stem coincides with the imaginary rotation axis of the symmetrical region. Hence, the tRNA translocation involves two correlated motions: mRNA/tRNA shift and a rotation of the tRNA single-stranded aminoacylated-3′ end (figure 2*a*). This rotatory motion is navigated and guided by the ribosomal architecture, which provide the scaffold of a pattern composed of essential nucleotides [19].

This motion results in stereochemistry optimal for peptide bond formation and in geometry ensuring that nascent proteins entrance into their exit tunnel. The rotatory passage of the A-site tRNA 3′ end into the P-site allows the creation of the reaction transition state (figure 2*b*) [18], vacates the space at the A-site for the entrance of the next aminoacylated tRNA and results in the entrance of the 3′ end of the A-site tRNA into the P-site, thus assisting the release of the P-site leaving group and ensuring the processivity of protein biosynthesis. It is worth noting that the rotatory motion positions the proximal 2′-hydroxyl of P-site tRNA A76, which is involved in substrate-mediated acceleration [20], in the same position and orientation found in crystals of the entire ribosome with mRNA and tRNAs, as determined independently in two laboratories [6,7].

## 3. An RNA apparatus for chemical bonding

Within the contemporary ribosome, this entity has a pocket-like structure, with a shape that seems to be capable of maintaining stable conformation as an independent chemical entity with ribozyme catalytic capabilities (figures 1*c* and 2*c*). Hence it appears to be a relic of an ancient ribozyme that was capable of catalysing various RNA-involved reactions, which was adopted by the amino acids once they appeared and invaded the RNA world. Then it could have evolved into a molecular machine for peptide bond formation and non-coded amino acid polymerization, which was later optimized to become a pocket in which each half hosts a slightly different substrate.

The backbone fold of each half of this semi-symmetrical region resembles motifs identified in various ‘ancient’ as well as ‘modern’ natural RNA molecules of comparable size, consisting mainly of stem-elbow-stem motifs. Examples are tRNA, gene regulators, riboswitches, RNA polymerases, ribozymes catalysing phosphodiester cleavage, RNA processing and RNA modification. Many of these ribozymes are also believed to be remnants from the prebiotic world, which are supposed to be sufficiently stable to survive environmental alterations and evolutionary stresses.

It was shown recently that certain RNA bases could have been produced under prebiotic conditions [21]. Additionally, it was shown that RNA can replicate itself [22–24] and ribozyme-catalysed transcription of an active ribozyme from an RNA template has been demonstrated [25]. Hence, a dimeric RNA pocket could have been formed from two self-folded RNA chains of identical, similar or different sequences, either spontaneously or by gene duplication or gene fusion. Dimerization in a pseudo-symmetrical manner could have occurred spontaneously, by using surface complementarity obtained by tertiary interactions (e.g. the common GNRA tetra loop (where G is guanine, N is any nucleotide, R is any purine and A is adenine) and the abundant and ubiquitous ‘A-minor’ structural motif), or assisted by other molecules acting as small chaperones that offer stabilization. According to our hypothesis, this may have been the way the ancient bonding machine was formed. Thus, the suggested proto-ribosome could have been constructed from the two symmetry related ribosomal core units, each composed of about 60–90 nucleotides forming two helices connected via an elbow, namely a single-stranded region.

Activated amino acids could be the suitable substrates of the ancient machine. These could be formed by their attachment to nucleotides, exploiting rather common reactions that were shown to occur by diverse processes [26–29]. Hence mono, di- and tri-nucleotides could carry the amino acids to the bonding pocket-like entity. Previous studies indicated that the suggested pocket-like entity (namely the proto-ribosome) could have accommodated amino acids bound to up to three nucleotides [13–17].

Conceptually, the initial dipeptides could be elongated and form oligopeptides via the same reaction principles. The existence of well-performing polypeptides, catalysing fundamental reactions and/or stabilizing the machine producing them, may have led to the emergence of the genetic code. It is conceivable that initially short oligopeptides were produced accidentally. Among them, those that could provide additional structural support for the synthetic apparatus that produced them could attach to it and form structural arrangements similar to protein–RNA interactions of the contemporary ribosome. According to this suggestion, the proto-ribosome produced peptides with amino acid composition that was sufficiently biased for fulfilling simple tasks, giving a selective advantage.

We are assessing the feasibility of the existence of a dimeric proto-ribosome capable of the formation of chemical bonds according to the following scheme. First, we are assessing the tendency of various RNA chains to dimerize and form a pocket-like entity (figures 1*c* and 2*c*). Pocket formation by dimerization is supported by the finding that mutations in the regions involved in A/P interaction (figure 2*c*) within the contemporary ribosome prevented dimerization. A noteworthy example is the segment introduced by us into the construct that is composed of the GNRA tetra loop containing an A-minor interaction (A-minor interactions involve adenine and the minor groove of an RNA double helix) that seems to stabilize the pocket.

Among the various RNA segments that have been synthesized *in vitro*, several, albeit not all chains with sequences resembling those observed in the contemporary ribosome, formed dimers that may adopt a ‘pocket-like’ structure. So far, a marked preference for dimerization was detected for sequences resembling the P-region of the PTC, including those that underwent so-called ‘mutational’ alterations by *in vitro* mutagenesis, introduced in the regions that connect the two symmetrical halves of our constructs (figure 2*c*). Remarkably, P-loop incorporation (figure 2*d*) into the RNA constructs did not prevent dimerization. These dimers are tested for their ability to bind small molecules, nucleotides and amino acids conjugated with short nucleotides. Preliminary results indicated detectable binding of several compounds representing RNA world components.

**Figure 3.**
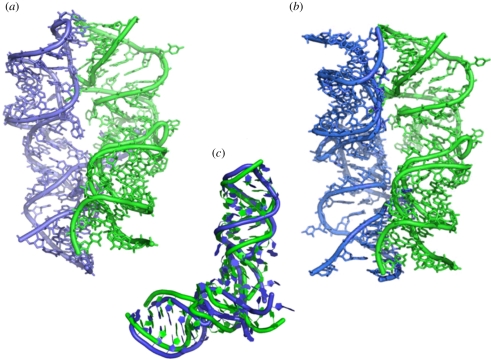
The similarities and differences between the A- and the P-sites. In all panels, the A-site and P-site tRNAs are shown in blue and green, respectively. (*a*) View into the symmetrical region as appears within the contemporary ribosome. (*b*) View from a direction similar to that shown in (*a*) of the predicted structure of a construct made of two P-regions. One of them is in its native position, and the second superposed on the native position of the A-region, thus mimicking the suggested ‘all P-region proto-ribosome’. (*c*) Superposition of the A- and P-regions in the contemporary ribosome, highlighting the differences between them, which may have resulted from evolving optimization.

So far, particularly noticeable preference to dimerize was observed for sequences identical to or resembling the P-region of the PTC, contrary to those that are similar to the A-site region. This unexpected observation may indicate that the precursor of the ancient ‘pre-proto-ribosome’ machine was built mainly by the dimerization of the P-portion of the symmetrical region. The reasons for this preference are still to be clarified. It may result from a higher structural, chemical or functional stability. This preference may indicate that the proto-ribosome (or the pre-proto-ribosome) was composed of a dimer of the P-region (figure 3*a*,*b*), which is in accord with the contemporary accommodation of the initial tRNA at the P-site of the PTC. Similar preference was observed for semi-random-sequence constructs of size and base pair composition with potential to self-fold into conformations resembling the ribosome symmetrical region, presumably owing to the above considerations [13–17]. The non-uniform tendency to dimerize of selected RNA sequences over very similar, though not identical, ones indicates that survival of the fittest and natural selection seemed to play a major role in the prebiotic world, although these properties are commonly related to the evolution of species. Regardless of the reason, the above findings point to gene duplication as the preferred pathway of the natural production of the proto-ribosome.

The emergence of coded translation that includes the growing complexity of the ribosome into the size and shape allowing programmed translation could have occurred as a consequence of the survival of those oligopeptides that were produced accidentally, but became useful in the RNA world. Their existence could have been the driving force for the production of some kind of replication machinery. Coevolution of the proto-ribosome into the modern complex molecular machine may have led to the small, albeit significant, differences between the A- and the P- regions (figure 3*c*) dictating the involvement of carriers that could decode while bound to the cognate amino acid, namely the tRNA molecules, similar to the opinion discussed by Di Mauro [30]. This suggestion is in accord with the results of the analysis of the intra-RNA interactions within the ribosome [31] as well as with the conclusion of a study in which the ribosome was examined from the opposite direction, namely from the surface into the core [32]. Further support for the existence of a proto-ribosome as a pocket-like bonding machine that can host two substrates required for peptide bond formation (that later evolved into the aminoacylated and peptidylated tRNAs) is provided by the finding that, in contrast to the very high conservation of the A- and P-sites, variability in the shape and the environment of the site of the exiting tRNA molecule (E-site) have been observed [33].

## 4. Conclusions

Here, we suggest that a vestige of an RNA apparatus with ribozyme capabilities is embedded and functions within the modern ribosome. We advocate the existence of a chemical prebiotic machine, originating from an oligonucleotide and proceeding via a self-folded unit into a self-assembled dimer, thus producing a chemical pocket that could turn into an apparatus for peptide bonds formation. Internal interactions could have stabilized it. Hence, it could have survived and functioned on its own. However, additional interactions, e.g. with its own products, non-coded oligopeptides, could have contributed to its stability. This hypothesis requires the existence of self-replicating RNA molecules that can fold and create a pocket with catalytic capabilities.

In short, the ribosome's architecture hints at its evolutionary pathway. Thus, it seems that the proto-ribosome was originally an RNA dimeric machine for performing RNA needs prior to the appearance of amino acids. The amino acids snatched it and turned it into an efficient machine producing proteins. Originally, small oligopeptides were formed. Those found to be useful have survived and led to the creation of a mechanism for duplicating themselves. This suggests that the more fit proto-ribosome products guided the appearance of the genetic code, namely the genetic code was created according to its products.
